# Supplementary material for: Sulfate formation is dominated by manganese-catalyzed oxidation of SO2 on aerosol surfaces during haze events
Source: Nat Commun. 2021 Mar 31;12:1993. doi: 10.1038/s41467-021-22091-6 (PMC8012371; doi:10.1038/s41467-021-22091-6)
Supplement: Supplementary file 1 — Supplementary Information [file 41467_2021_22091_MOESM1_ESM.pdf]

Supplementary Information for

Sulfate formation is dominated by manganese-catalyzed oxidation of SO_2 on aerosol surfaces during haze events

Weigang Wang[1, 2, 11], Mingyuan Liu[1, 2, 11], Tiantian Wang[3,11], Yu Song[3]*, Li Zhou[1], Junji Cao[4], Jingnan Hu[5], Guigang Tang[6], Zhe Chen[7], Zhijie Li[8], Zhenying Xu[3], Chao Peng[1], Chaofan Lian[1], Yan Chen[1], Yuepeng Pan[8], Yunhong Zhang[7], Yele Sun[8], Weijun Li[9], Tong Zhu[3], Hezhong Tian[10], Maofa Ge[1, 2] *

[1] State Key Laboratory for Structural Chemistry of Unstable and Stable Species, Beijing National Laboratory for Molecular Sciences (BNLMS), CAS Research/Education Center for Excellence in Molecular Sciences, Institute of Chemistry, Chinese Academy of Sciences, Beijing, China.

[2] University of Chinese Academy of Sciences, Beijing, China

[3] State Key Joint Laboratory of Environmental Simulation and Pollution Control, Department of Environmental Science, Peking University, Beijing, China.

[4] Key Laboratory of Aerosol Chemistry and Physics, State Key Laboratory of Loess and Quaternary Geology, Institute of Earth Environment, Chinese Academy of Sciences, Xi'an, China.

[5] Institute of Atmospheric Environment, Chinese Research Academy of Environmental Sciences, Beijing, China

[6] State Environmental Protection Key Laboratory of Quality Control in Environmental Monitoring, China National Environmental Monitoring Centre, Beijing, China

[7] The Institute of Chemical Physics, School of Chemistry and Chemical Engineering, Beijing

Institute of Technology, Beijing, China

[8] State Key Laboratory of Atmospheric Boundary Layer Physics and Atmospheric Chemistry, Institute of Atmospheric Physics, Chinese Academy of Sciences, Beijing, China

[9] Department of Atmospheric Sciences, School of Earth Sciences, Zhejiang University, Hangzhou, China

[10] State Key Joint Laboratory of Environmental Simulation & Pollution Control, School of Environment, Beijing Normal University, Beijing, China

[11] These authors contributed equally to this work.

*Corresponding authors. Email: gemaofa@iccas.ac.cn (M.G.); songyu@pku.edu.cn (Y.S.).

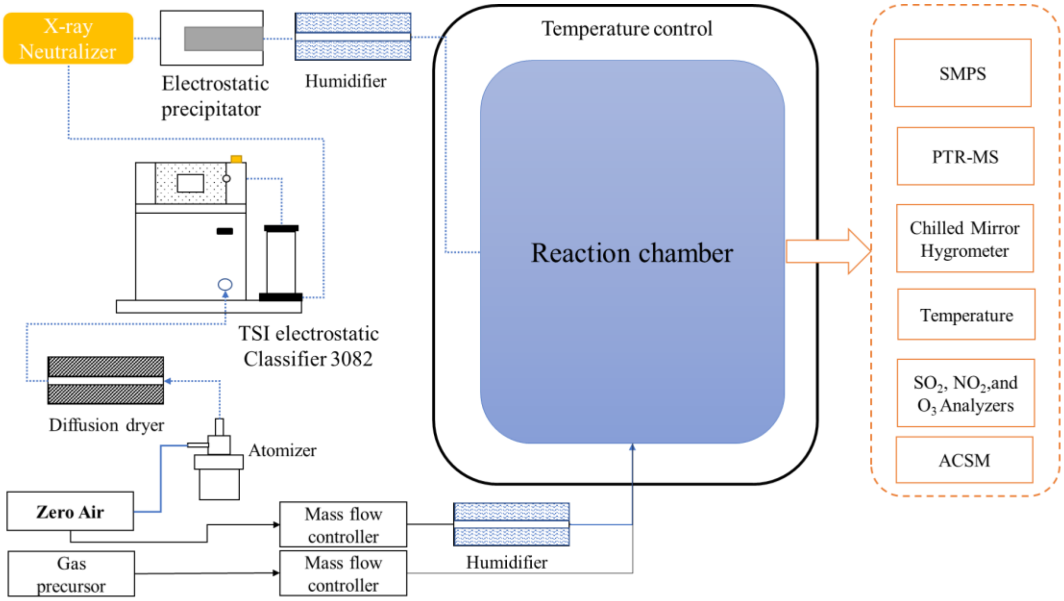

Supplementary Fig. 1. Schematic diagram of the reaction chamber used in this work along with the analytical methods used to detect reactants and products. SMPS, scanning mobility particle sizer spectrometer; PTR-MS, proton transfer reaction quadrupole mass spectrometry; ACSM, aerosol chemical speciation monitor. The schematic is not to scale.

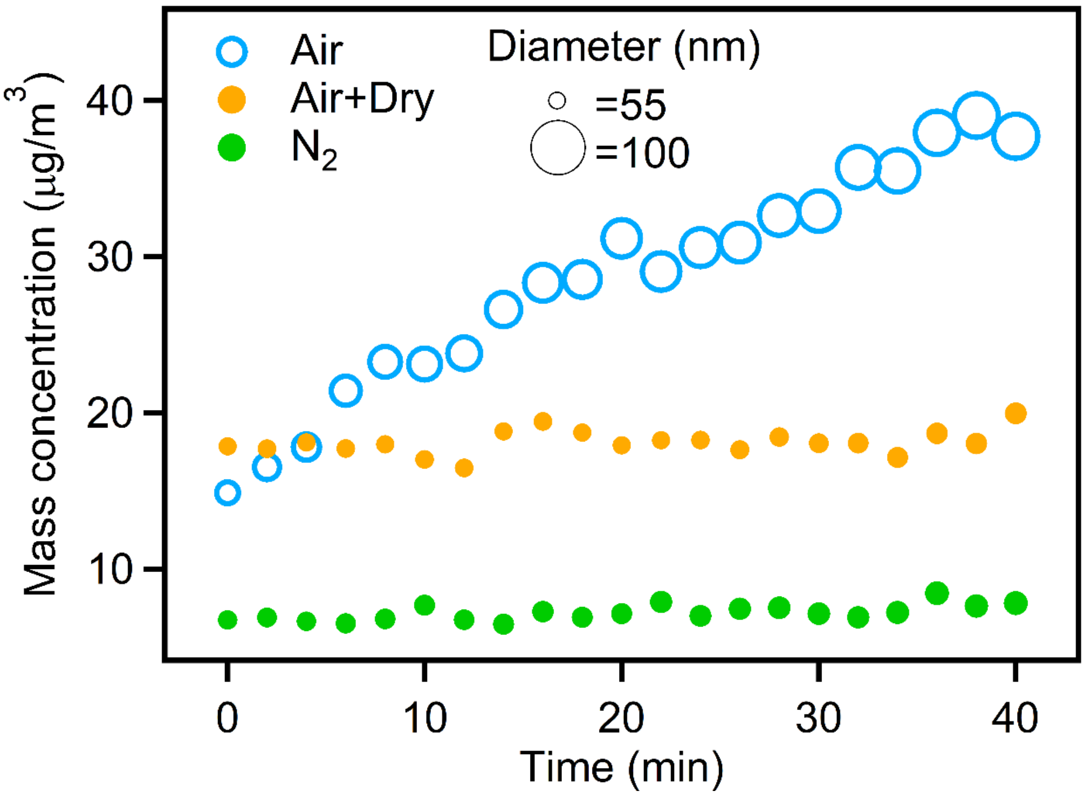

Supplementary Fig. 2. Measured temporal profiles of the particle mass concentration under different conditions at 298 K. In chamber experiments, ammonium sulfate particles containing 3.03% Mn^{2+} were exposed to SO_2 (116 ppb) and NH_3 (84 ppb) under relative humidity (RH) 89%. Blue open circles indicate the zero air condition; green solid circles indicate the N_2 condition; and orange solid circles indicate the dry air condition. The diameters of the circles indicate particle diameter.

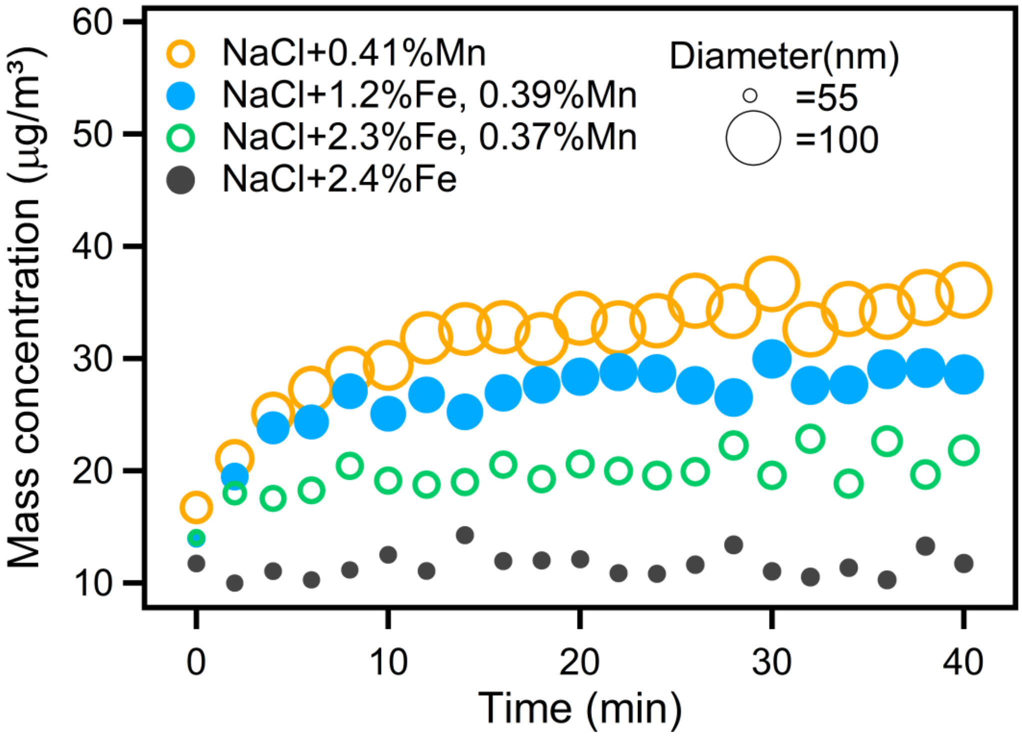

Supplementary Fig. **3**. Temporal profiles of the particle mass concentration measured under different conditions at 298 K. In the chamber experiments, NaCl particles containing different concentration of Fe^{3+} and Mn^{2+} were exposed to SO_2 (116 ppb) and NH_3 (84 ppb) under relative humidity (RH) 89%.

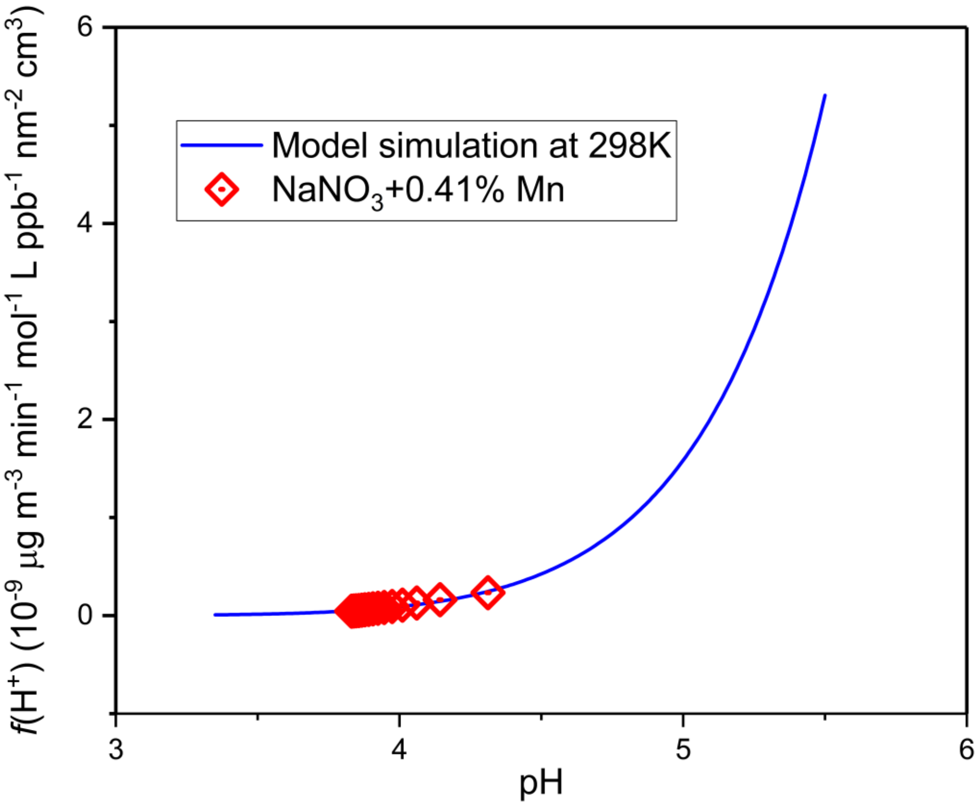

Supplementary Fig. 4. Reaction rate of a sodium nitrate seed particle at 298 K compared to model simulation (equation 4–7). In chamber experiments, sodium nitrate particles containing different concentration of 0.41%Mn^{2+} were exposed to SO_2 (116 ppb) and NH_3 (84 ppb) under relative humidity (RH) 89%. There was no obvious reaction rate decrease in $NaNO_3$ seed reaction compared to the model (Fig. 3a, Method equation 4–7).

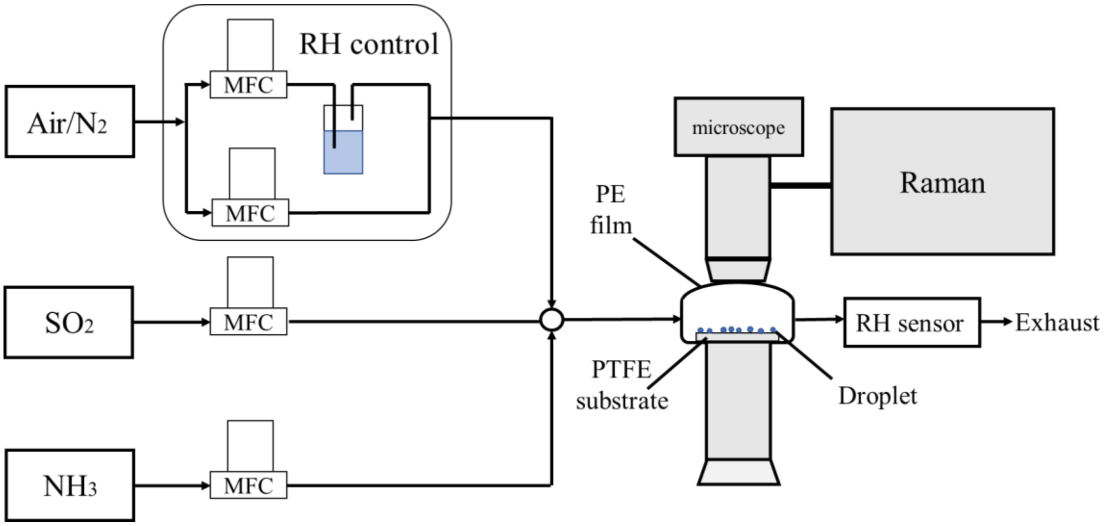

Supplementary Fig. **5**. Schematic of the micro-Raman experiments. MFC represents mass flow controller, PE represents polyethylene, and PTFE represents polytetrafluoroethylene.

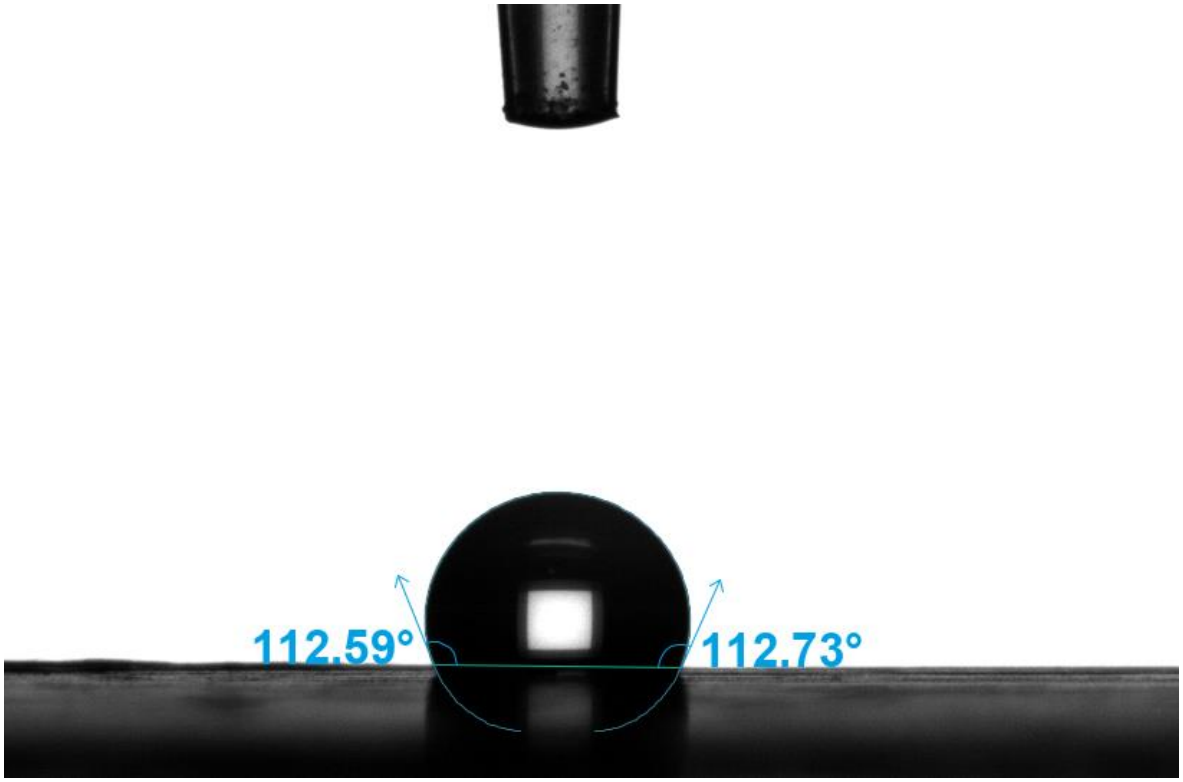

Supplementary Fig. **6**. Contact angle of a 3 mol L^{-1} NH_4Cl droplet on polytetrafluoroethylene (PTFE) substrate.

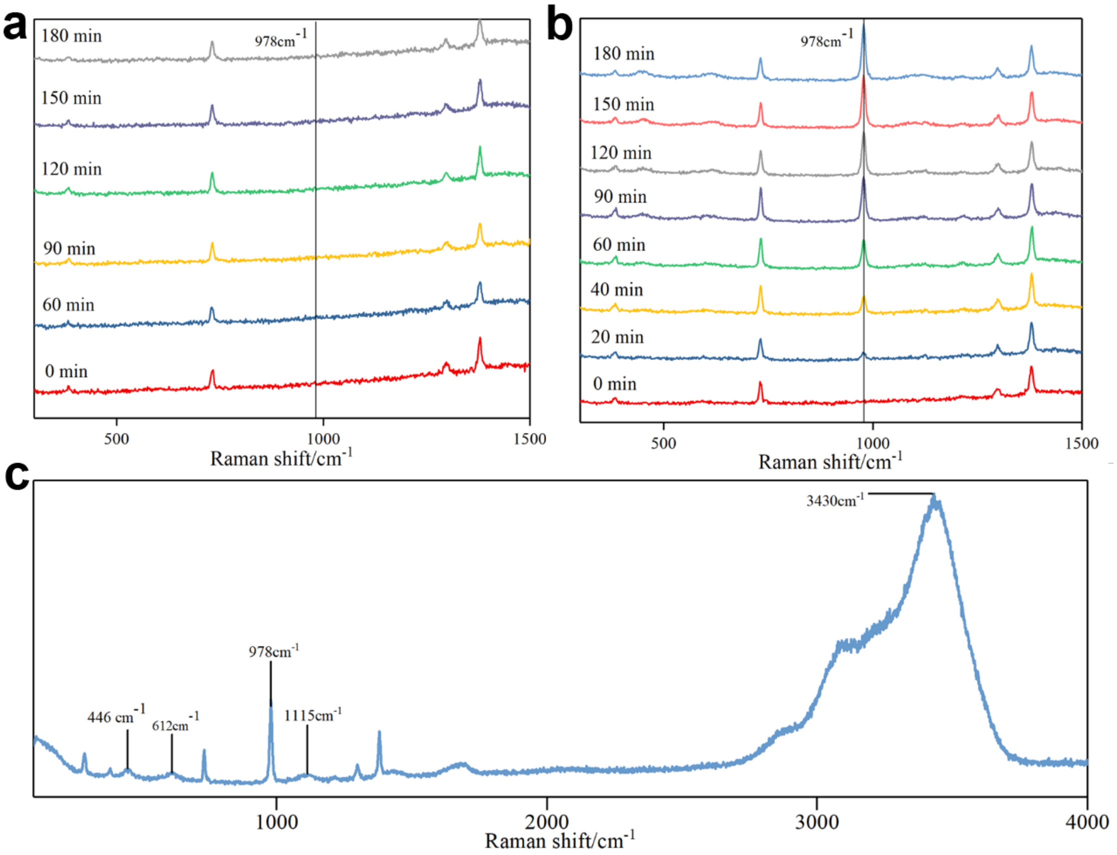

Supplementary Fig. 7. Raman spectra for two sets of experiments performed under different conditions. **a**, 3 M NH_4Cl with 0.03 M $MnCl_2$ droplets was exposed to 327 ppbv SO_2/N_2 at a flow rate of 1 L/min under 85 ± 1% relative humidity (RH); the droplet diameter was 10 µm. **b**, Zero air was used as the carrier gas. **c**, Raman spectrum of NH_4Cl+$MnCl_2$ droplet after a 180-min reaction with 327 ppbv SO_2/zero air.

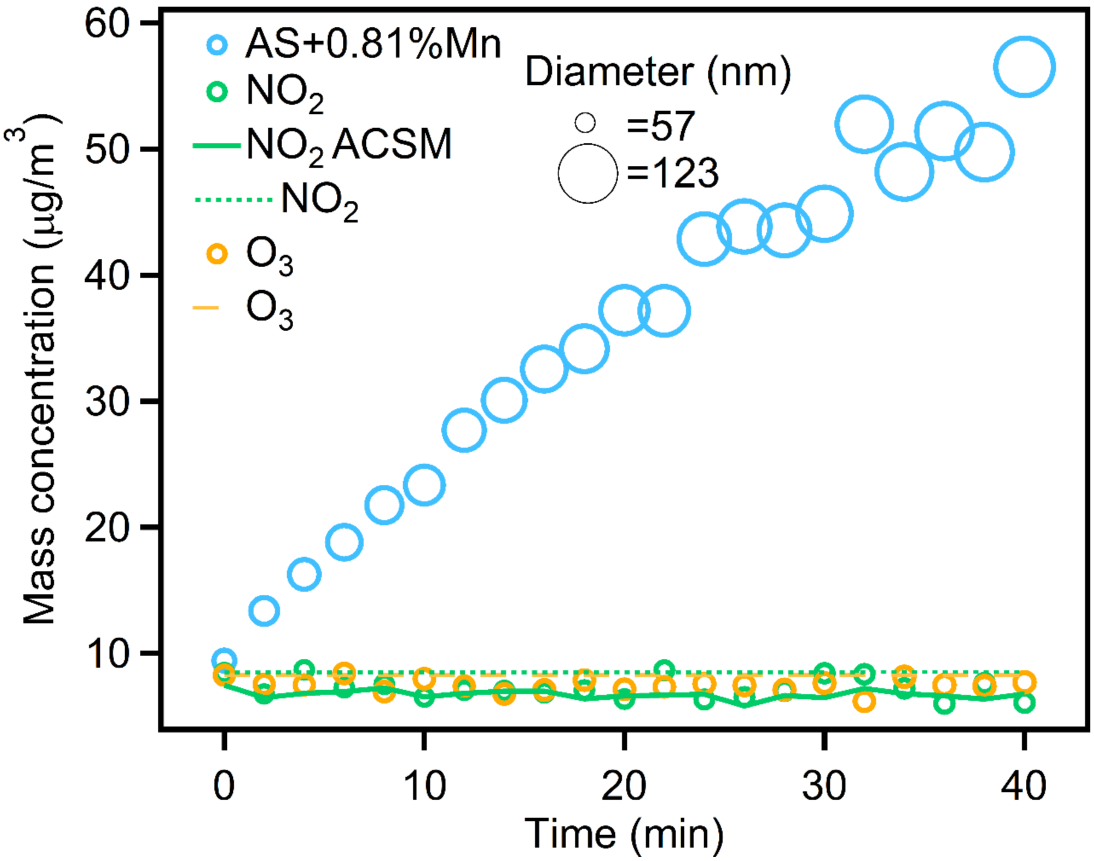

Supplementary Fig. 8. Sulfate formation from NO_2 and O_3 in chamber experiments conducted at 278 K. Blue open circles correspond to the chamber experiment with $(NH_4)_2SO_4$ seed particles containing 0.81% Mn^{2+}, 116 ppbv SO_2, and 84 ppbv NH_3 under 83% relative humidity (RH); green open circles correspond to the chamber experiment with $(NH_4)_2SO_4$ particles exposed to 230 ppbv NO_2, 116 ppbv SO_2, and 98 ppbv NH_3 under 82% RH; yellow open circles are the chamber experiment with $(NH_4)_2SO_4$ particles exposed to 228 ppbv O_3, 116 ppbv SO_2, and 98 ppbv NH_3 under 83% RH. The green line is the ACSM measurement result. Circle diameter indicates particle diameter. The dashed line depicts the change in mass concentration for the same initial chamber conditions calculated with aqueous phase kinetic parameters. Due to the lack of E/R value, the rate constant (k_{high}) was used for calculating the NO_2 pathway. Details are given in Table S2.

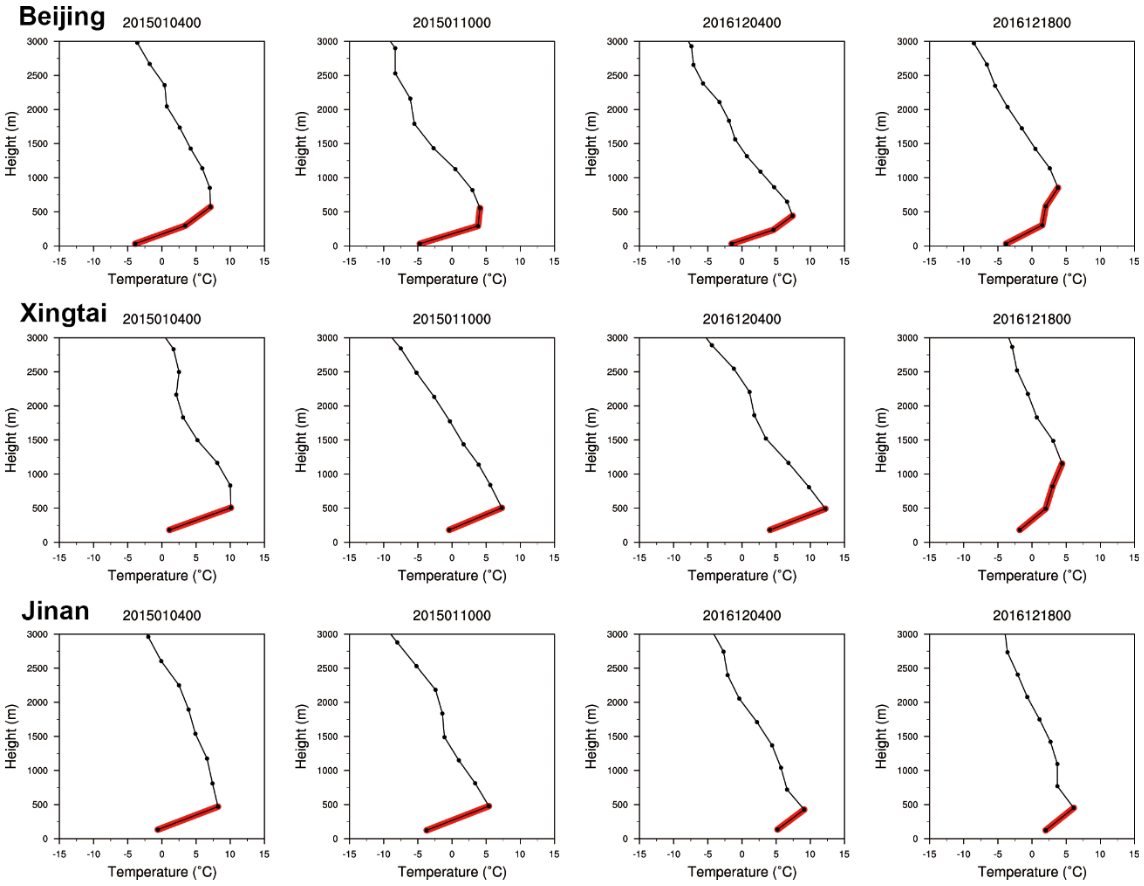

Supplementary Fig. **9**. Observed vertical temperature profile at 00 UTC (Coordinated Universal Time) on January 4 and 10, 2015, and December 4 and 18, 2016 at Beijing, Xingtai, and Jinan. The temperature inversions are marked with red lines.

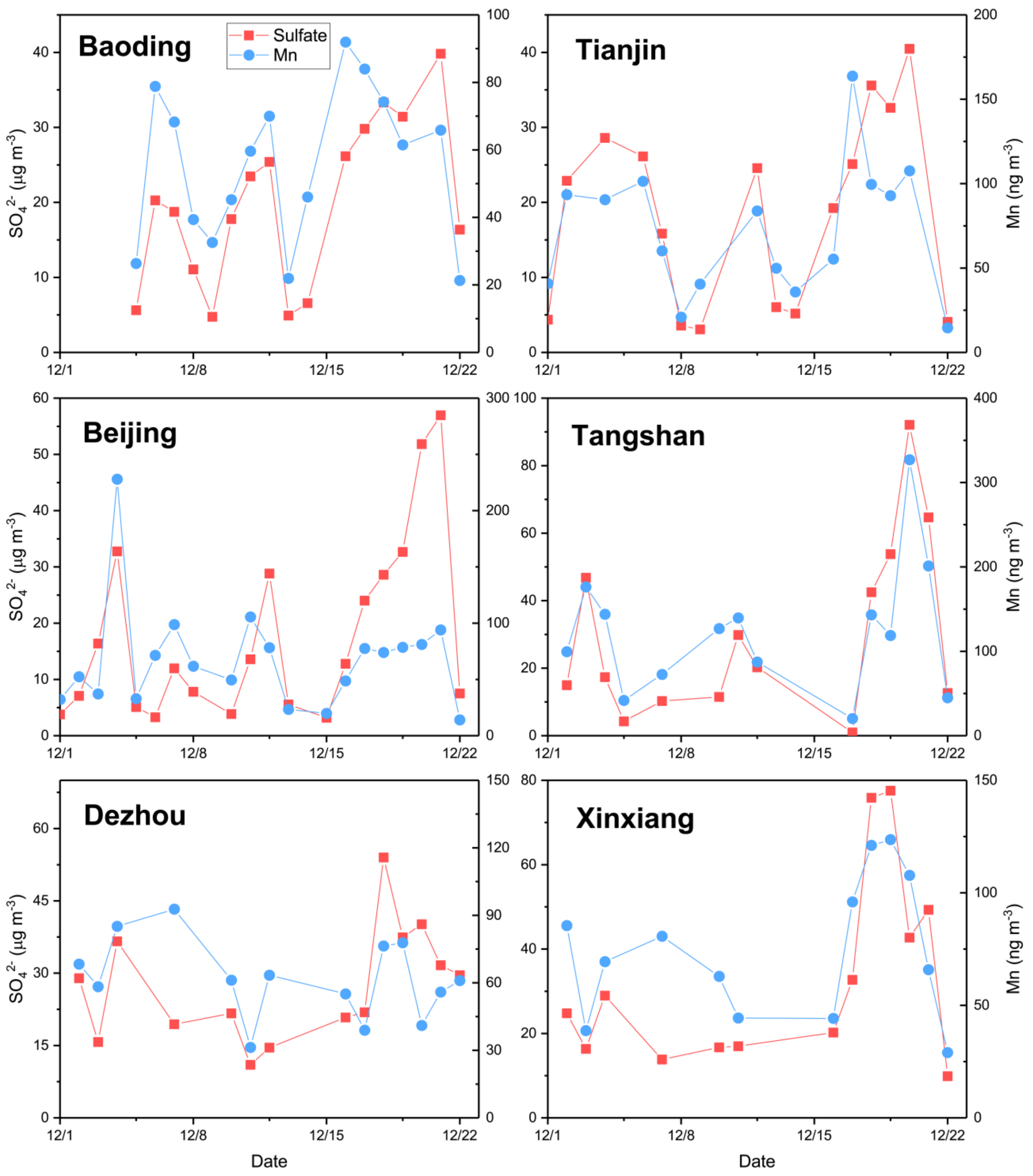

Supplementary Fig. **10**. Temporal variation of sulfate and Mn concentration in $PM_{2.5}$ at different sampling sites in NCP (Baoding, Tianjin, Beijing, Tangshan, Dezhou, and Xinxiang) in December 2016. Blue circles refer to Mn (right coordinate axis), Red squares refer to sulfate (left coordinate axis).

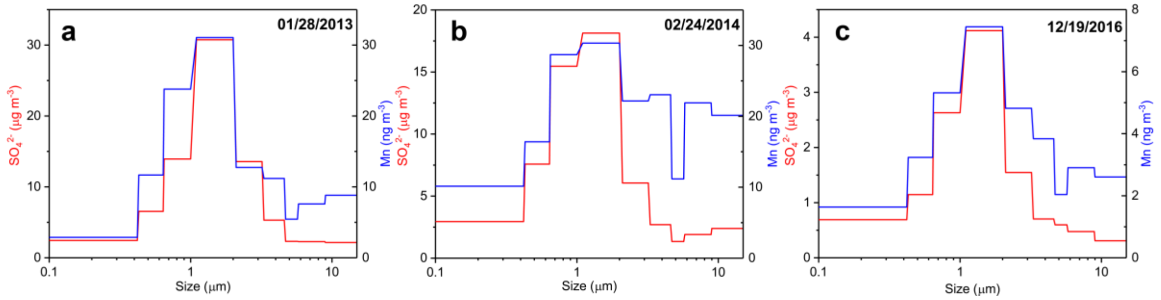

Supplementary Fig. 11. Size distributions of sulfate and Mn in aerosol in Beijing. Sampling periods: (a) Jan. 28th–29th, 2013, (b) Feb. 24th–26th, 2014, and (c) Dec. 19th–20th, 2016. The red line represents sulfate content; the blue line represents Mn content.

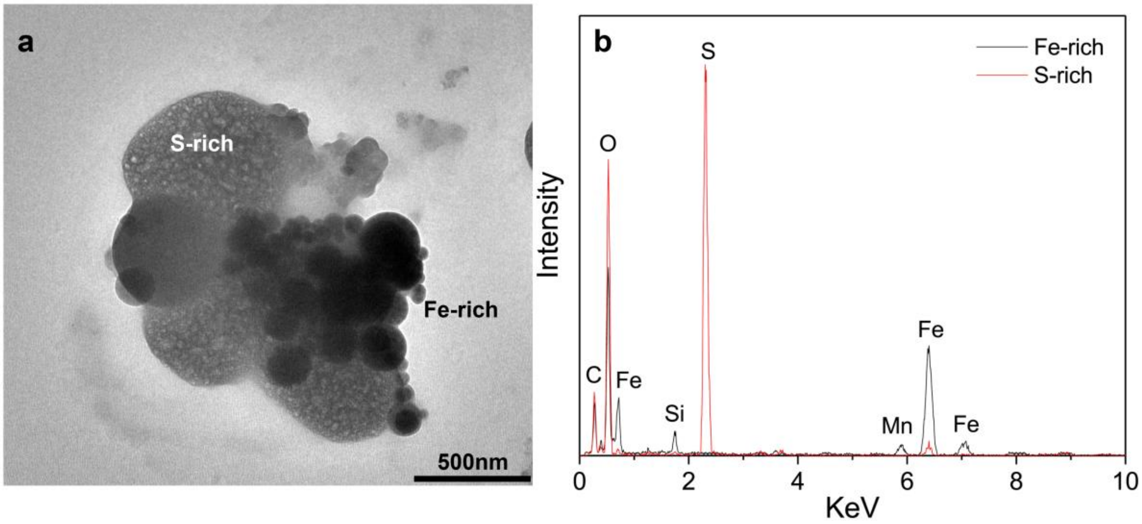

Supplementary Fig. 12. Morphology and element distribution of a single particle sample collected in urban Beijing, Dec. 30th, 2017. (a) TEM image of sample, (b) EDX (energy dispersive X-ray analysis) spectrum of the S-rich part (red line) and the Fe-rich part (black line). The sampling site was located at the China University of Mining and Technology-Beijing.

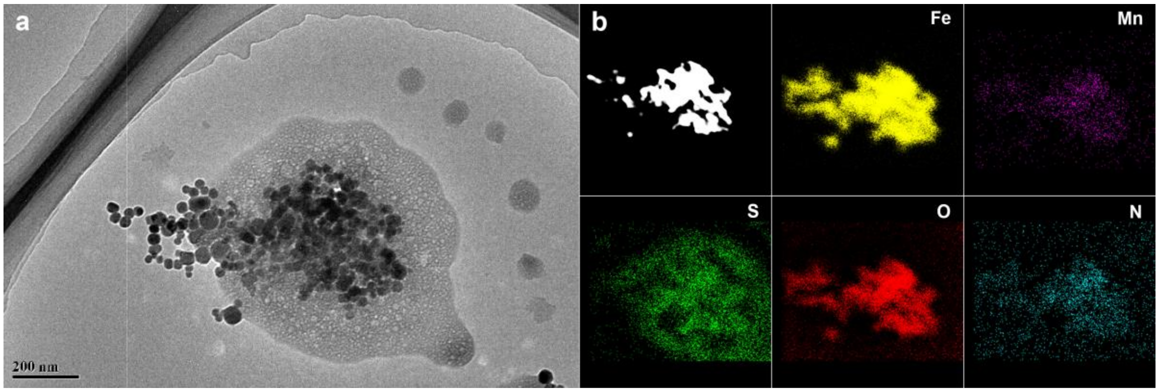

Supplementary Fig. 13. TEM image and element maps of a single particle sample collected in Shijiazhang (Hebei Province), Oct. 19th, 2020. (a) refers to the morphology of the sample. (b) refers to the element mapping of the sample. The sample located was the Hebei Normal University.

130

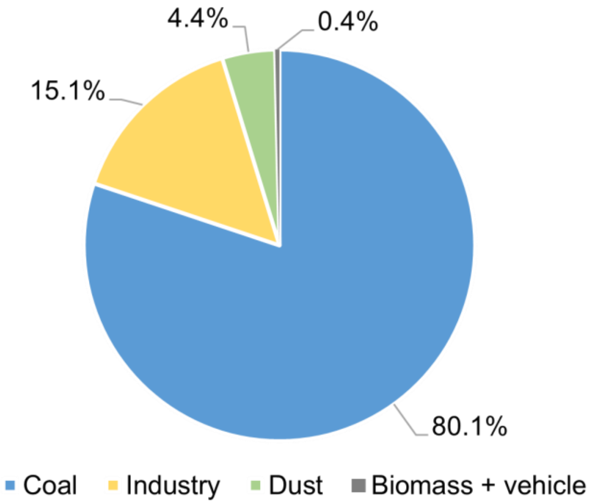

Supplementary Fig. 14. Source contributions of Mn in North China Plain during haze events from Dec. 1st to 22nd, 2016 by using ambient $PM_{2.5}$ chemical compositions and positive matrix factorization method. The sources include coal combustion, industrial process, soil dust, biomass burning and vehicle emission.

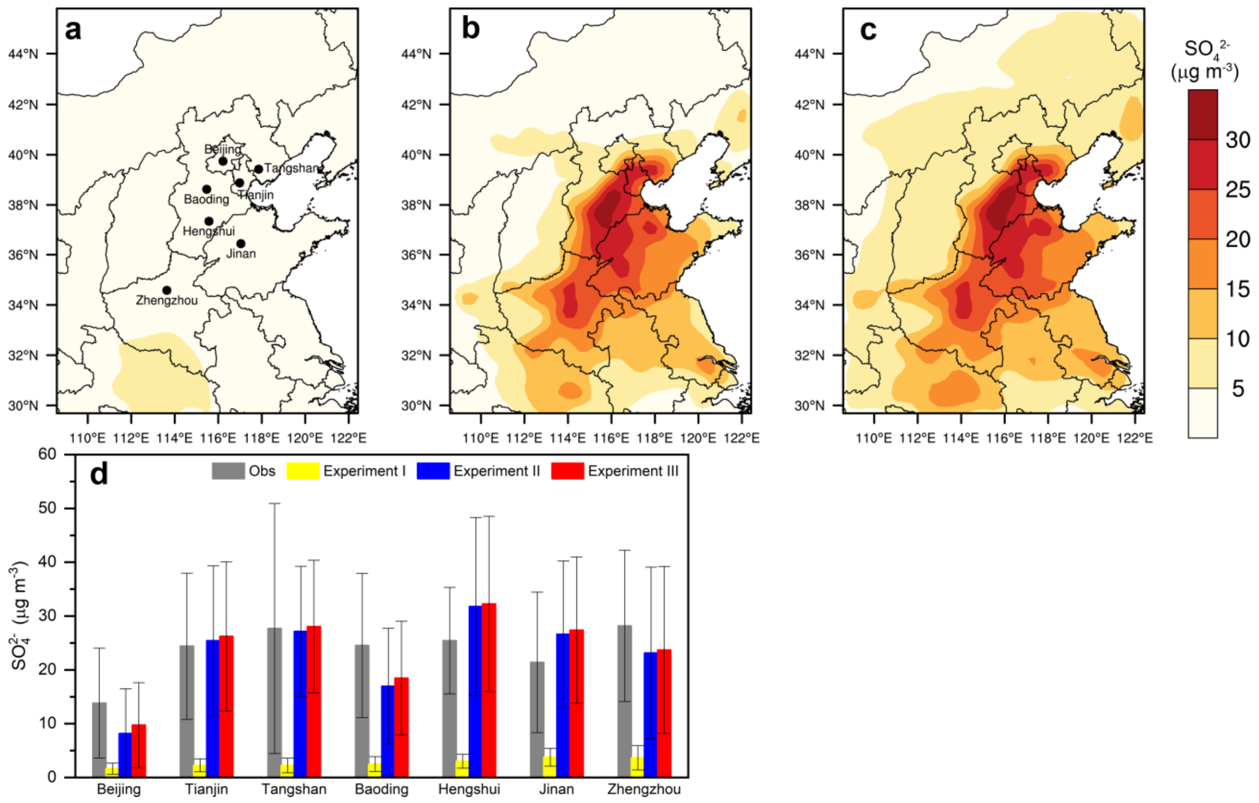

Supplementary Fig. 15. Observed and WRF-Chem-simulated sulfate concentrations. **a**, Spatial distributions of the WRF-Chem-simulated sulfate concentration in Experiment I during the three haze episodes conducted in January 2015. **b**, The same as (a), but for Experiment II. **c**, The same as (a), but for Experiment III. **d**, Comparison of the observed and WRF-Chem-simulated sulfate concentration during the three haze episodes in January 2015, at the seven sites marked by black dots in (a). Error bars represent standard deviation. Maps were created by the authors using NCAR Command Language software Version 6.6.2.

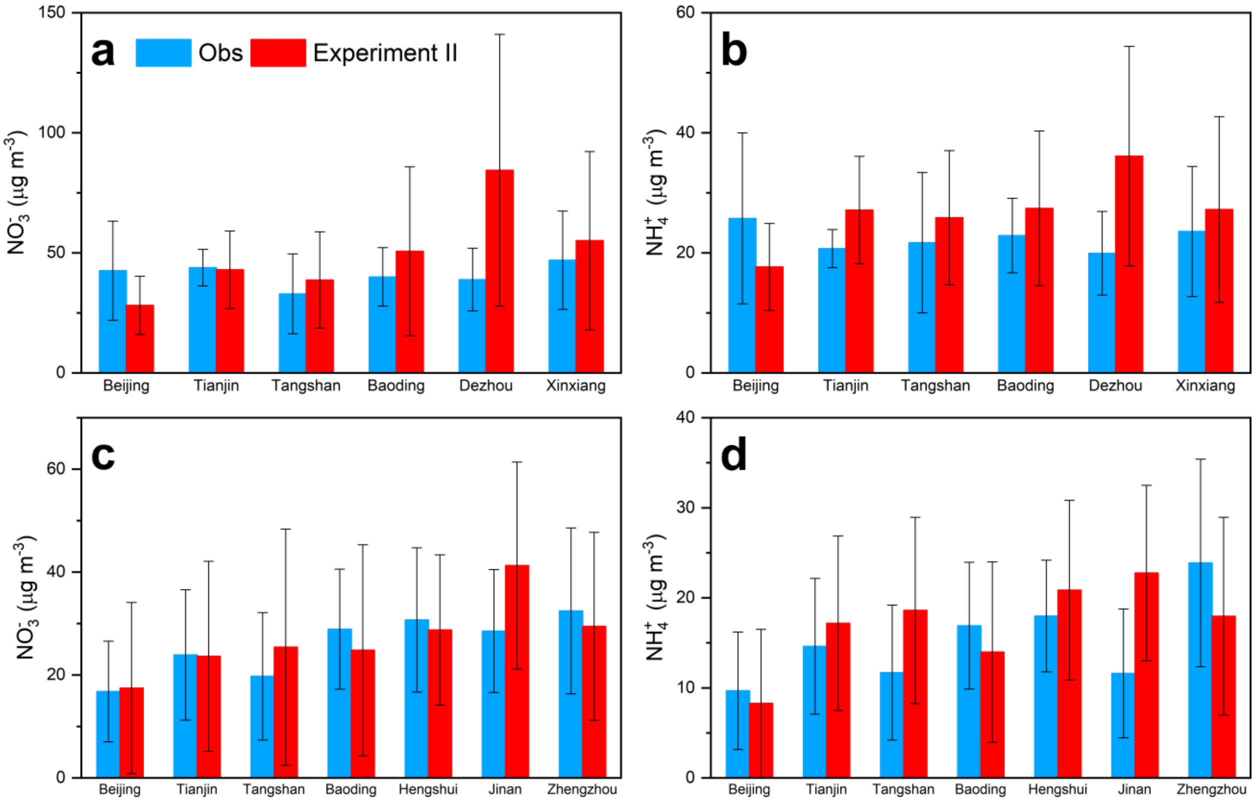

Supplementary Fig. **16**. Comparison of the observed and WRF-Chem-simulated (Experiment II) concentrations of NO_3^-, NH_4^+. **a**, Comparison of the observed and WRF-Chem-simulated NO_3^- during the three haze episodes in December 2016 at the six sites marked by black dots in Fig. 6a. **b**, The same as (a), but for NH_4^+. **c**, The same as (a), but for the three haze episodes in January 2015 at the seven sites marked by black dots in Supplementary Fig. 15a. **d**, The same as (c), but for NH_4^+. Error bars represent standard deviation.

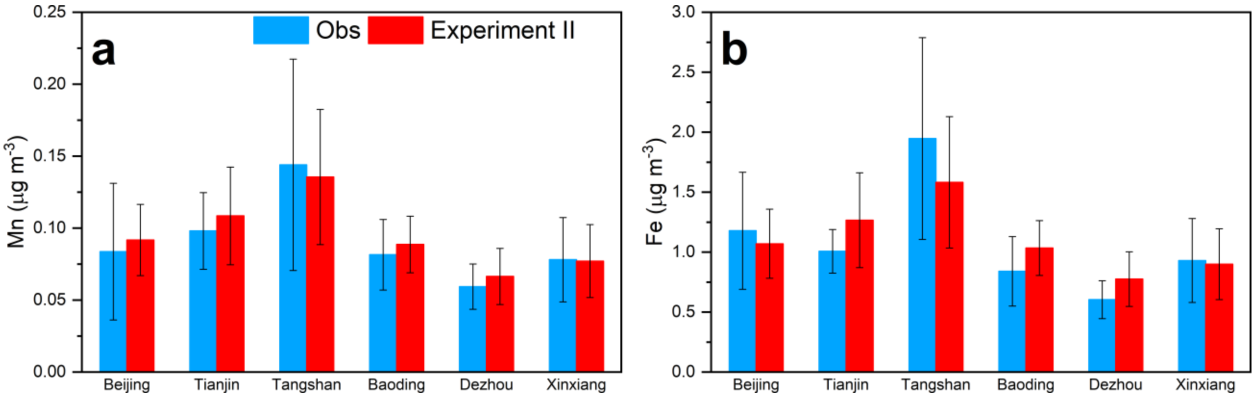

Supplementary Fig. **17**. Comparison of the observed and WRF-Chem-simulated (Experiment II) (a) Mn and (b) Fe concentrations during the three haze episodes in December 2016 at the six sites (marked by black dots in Fig. 6a). Error bars represent standard deviation.

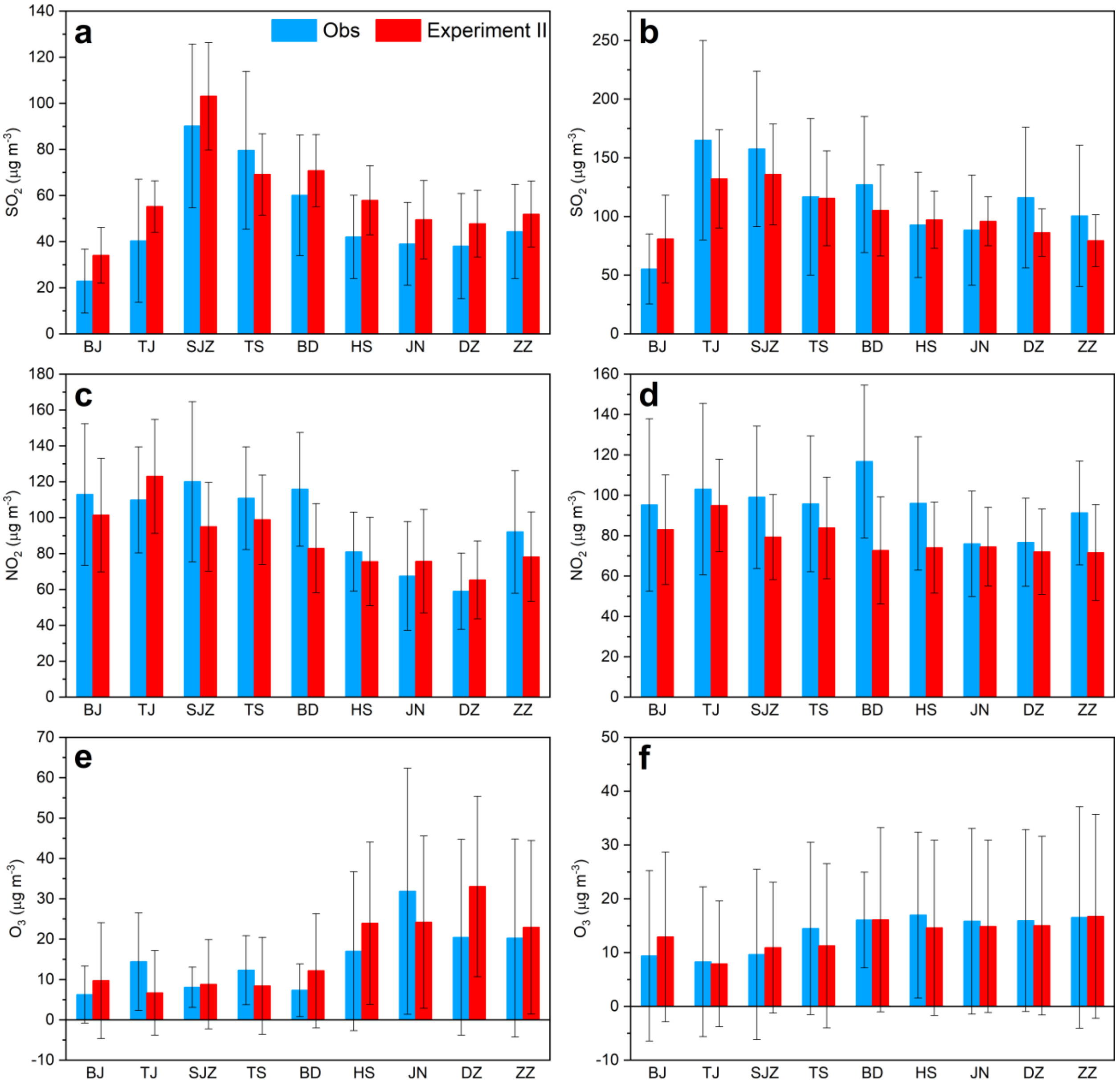

Supplementary Fig. **18**. Comparison of the observed and WRF-Chem-simulated (Experiment II) concentrations of SO_2, NO_2, and O_3 at nine sites (marked by black dots in Supplementary Fig. 20). **a**, Comparison of the observed and WRF-Chem-simulated SO_2 during the three haze episodes in December 2016. **b**, The same as (a), but for the three haze episodes in January 2015. **c**, The same as (a), but for NO_2. **d**, The same as (b), but for NO_2. **e**, The same as (a), but for O_3. **f**, The same as (b), but for O_3. Error bars represent standard deviation.

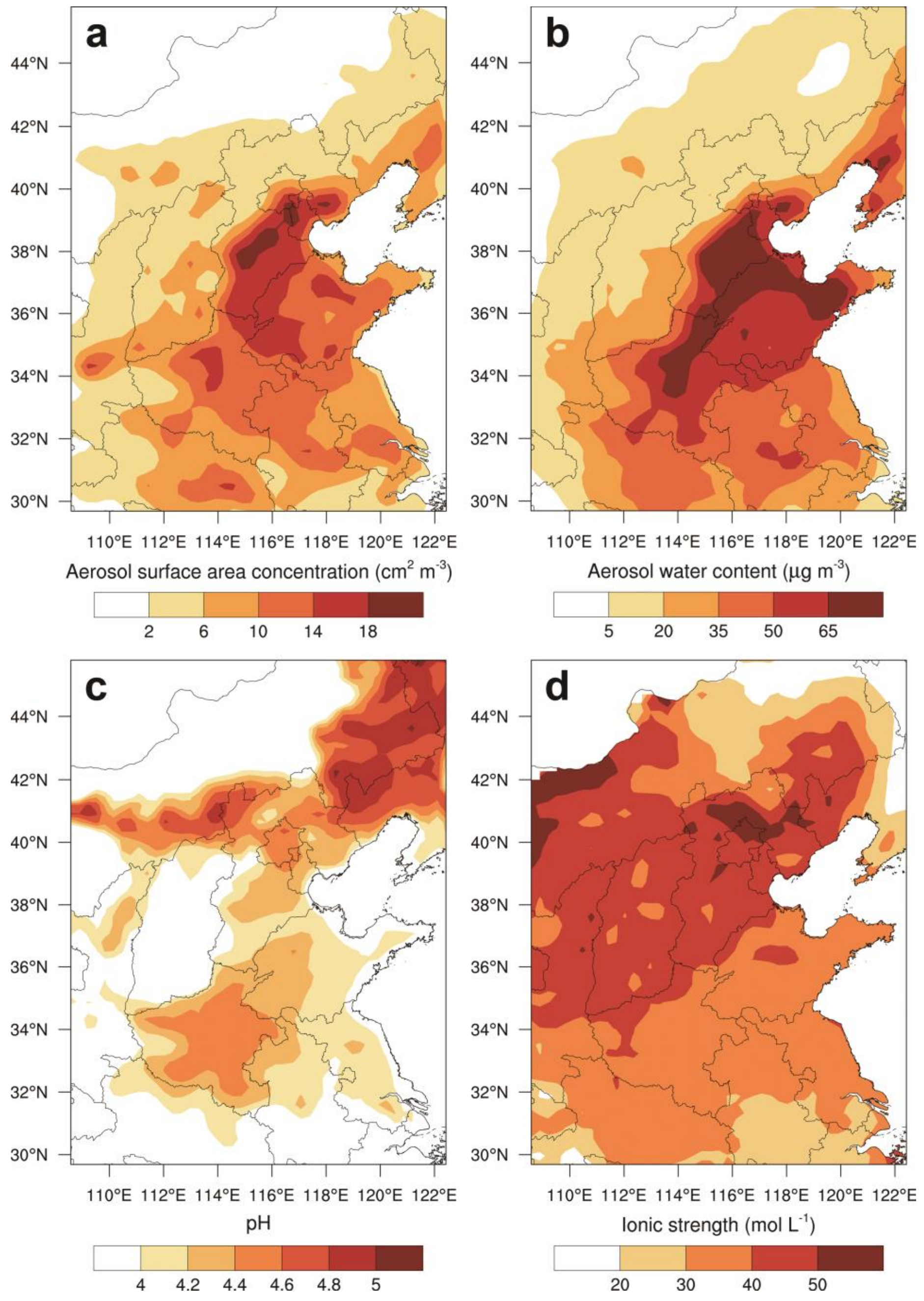

Supplementary Fig. **19**. Spatial distributions of the WRF-Chem-simulated (Experiment II). **a,** aerosol surface area concentration, **b,** aerosol water content, **c** aerosol pH, and **d** ionic strength during the six haze episodes during January 2015 and December 2016. Maps were created by the authors using NCAR Command Language software Version 6.6.2.

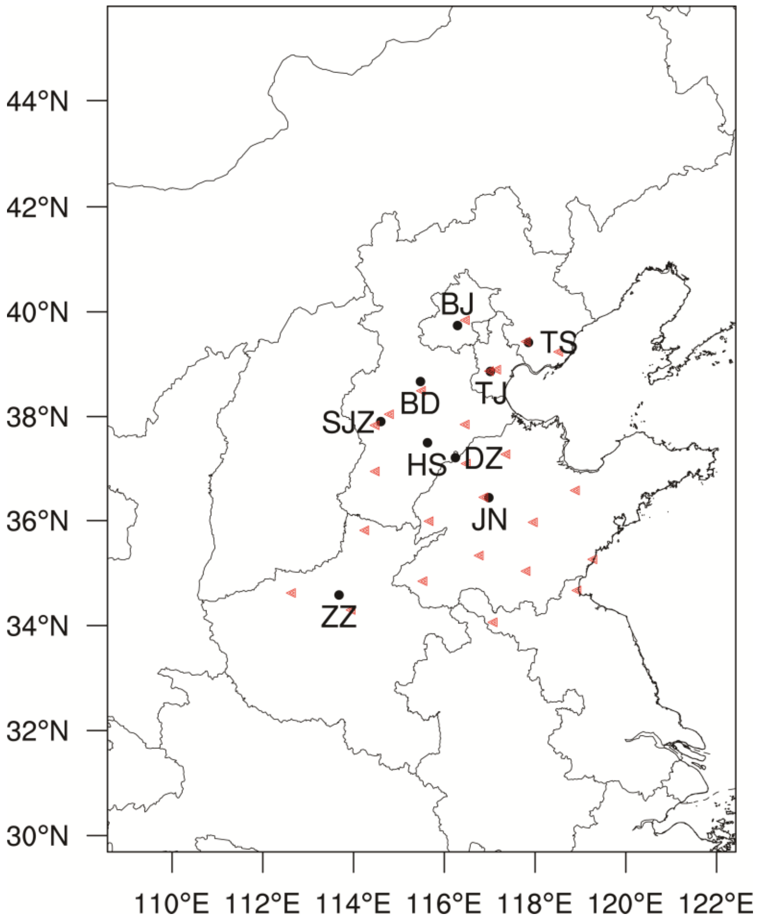

Supplementary Fig. 20. Spatial locations of the observation stations of gas phase species (black dots) and meteorological parameters (red triangles). BJ: Beijing, TS: Tangshan, TJ: Tianjin, BD: Baoding, HS: Hengshui, DZ: Dezhou, JN: Jinan, SJZ: Shijiazhuang, ZZ: Zhengzhou. Maps were created by the authors using NCAR Command Language software Version 6.6.2.

Supplementary Table 1. Chamber experimental conditions

No.	Seed particle	SO_2 (ppbv)	NH_3 (ppbv)	Mn concentration (%)	RH (%)	T (K)	Other conditions
1	$(NH_4)_2SO_4$	116	84	3.03	89	298	
2	$(NH_4)_2SO_4$	116	84	1.59	89	298	
3	NaCl	116	84	0.41	80	298	
4	$(NH_4)_2SO_4$	116	84	0.81	89	298	
5	$(NH_4)_2SO_4$	180	84	3.03	89	298	
6	$(NH_4)_2SO_4$	60	84	3.03	89	298	
7	NaCl	116	84	0.41	89	298	
8	$(NH_4)_2SO_4$	116	84	5.54	89	298	
9	NaCl	116	84	0.41	89	298	
10	$(NH_4)_2SO_4$	116	84	0.81	81	298	
11	$(NH_4)_2SO_4$	116	84	3.03	87	298	N_2
12	$(NH_4)_2SO_4$	116	84	3.03	23	298	Dry
13	$(NH_4)_2SO_4$	116	84	5.60	88	298	TEM sample
14	$NaNO_3$	116	84	0.41	89	298	
15	NaCl	116	84	0	88	298	2.4%Fe
16	NaCl	116	84	0.37	88	298	2.3%Fe
17	NaCl	116	84	0.39	88	298	1.2%Fe
18	NaCl	116	84	0.42	84	288	
19	$(NH_4)_2SO_4$	116	84	1.59	88	288	
20	NaCl	116	84	0.81	89	288	
21	$(NH_4)_2SO_4$	116	84	0.82	82	288	
22	$(NH_4)_2SO_4$	116	84	0.82	81	288	
23	$(NH_4)_2SO_4$	116	84	0.82	85	288	
24	$(NH_4)_2SO_4$	116	56	0.82	87	283	
25	$(NH_4)_2SO_4$	116	97	0.82	81	283	
26	$(NH_4)_2SO_4$	116	70	0.82	84	283	
27	$(NH_4)_2SO_4$	116	90	0.82	83	283	
28	$(NH_4)_2SO_4$	116	84	0.81	86	278	
29	NaCl	116	84	0.41	87	278	
30	NaCl	116	84	0.41	82	278	

31	$(NH_4)_2SO_4$	116	84	0.41	82	278	
32	$(NH_4)_2SO_4$	116	42	0.81	95	278	
33	$(NH_4)_2SO_4$	116	49	3.24	93	278	
34	$(NH_4)_2SO_4$	116	90	0.82	83	278	
35	$(NH_4)_2SO_4$	116	90	0.82	82	278	
36	$(NH_4)_2SO_4$	116	84	0.81	84	278	
37	$(NH_4)_2SO_4$	116	97	0	83	278	O_3 228 ppb
38	$(NH_4)_2SO_4$	116	97	0	82	278	NO_2 230 ppb

Supplementary Table 2. Rate expression and ionic strength effects of the aqueous reactions

Oxidants	Rate expression	Rate constant	E/R (K)	I impact
NO_2 [3]	$k_1[NO_2(aq)][S(IV)]$	$k_{1,\,low}$= (0.14~2)×10^6 $M^{-1}s^{-1}$<br>$k_{1,\,high}$= (1.24~1.67)×10^7 $M^{-1}s^{-1}$		
H_2O_2 [6]	$\frac{k_2[H^+][HSO_3^-][H_2O_2(aq)]}{1+k_3[H^+]}$	k_2= 7.45×10^7 $M^{-1}s^{-1}$<br>k_3= 13 M^{-1}	4430	$\log\frac{k}{k^{I=0}} = -\frac{2A\sqrt{I}}{1+B\sqrt{I}}+2\beta I$ [1]<br>A=0.509$M^{-0.5}$;B=0.17$M^{-0.5}$<br>β=0.18M^{-1}
O_3 [2]	$(k_4[SO_2\cdot H_2O]+k_5[HSO_3^-]+k_6[SO_3^{2-}])[O_3(aq)]$	k_4= 2.4×10^4 $M^{-1}s^{-1}$<br>k_5= 3.7×10^5 $M^{-1}s^{-1}$<br>k_6= 1.5×10^9 $M^{-1}s^{-1}$	 5530<br>5280	$\frac{k}{k^{I=0}} = 1 + b_1 I$ [3,4]<br>b_1=1.94
Mn(II) [2]	$k_7[Mn(II)][S(IV)]$	k_7= 1000 $M^{-1}s^{-1}$<br>(for low S(IV))		$\log\frac{k}{k^{I=0}} = b_2\frac{\sqrt{I}}{1+\sqrt{I}}$<br>$b_2$=-4.07 [5]
Fe(III) [6,a]	pH≤3.0 $k_8[H^+]^{-1}[Fe(III)][S(IV)]$<br>3.0<pH≤4.5 $k_9[Fe(III)]^2[S(IV)]$<br>4.5<pH≤6.5 $k_{10}[S(IV)]$<br>pH>6.5 $k_{11}[S(IV)]$	k_8=6.0<br>k_9=1.0×10^9<br>k_{10}=1.0×10^{-3}<br>k_{11}=1.0×10^{-4}		
Fe(III)-Mn(II) [7]	pH≤4.2 $k_{12}[H^+]^{-0.74}[Mn(II)][Fe(III)][S(IV)]$<br>pH>4.2 $k_{13}[H^+]^{0.67}[Mn(II)][Fe(III)][S(IV)]$	k_{12}= 3.72×10^7 $M^{-1}s^{-1}$<br>k_{13}= 2.51×10^{13} $M^{-1}s^{-1}$		$\log\frac{k}{k^{I=0}} = b_3\frac{\sqrt{I}}{1+\sqrt{I}}$<br>$b_3$=-4.0 [3]
HCHO[1,8]	$(k_{14}[HSO_3^-]+k_{15}[SO_3^{2-}])[HCHO(aq)]$	k_{14}= 7.9×10^2 $M^{-1}s^{-1}$<br>k_{15}= 2.5×10^7 $M^{-1}s^{-1}$	4900<br>1800	[1]

[a] The rate expression of the Fe(III)-catalyzed reaction in Martin, et al. [6] was revised according to the source code of aqueous-phase chemistry module in WRF-Chem to make it applicable to the entire pH range.

Supplementary Table 3. Performance statistics for the meteorological predictions of WRF-Chem during haze episodes over the NCP.

	T2[a]	RH2[a]	WS10[a]	WD10[a]
Data pairs[b]	5815	5813	5345	4727
MeanObs[b]	2.2	64.9	1.9	188.0
MeanSim[b]	2.4	53.9	2.2	179.5
R[b]	0.9	0.7	0.5	0.3
MB[b]	0.3	-11.0	0.3	-8.5
RMSE [b]	2.5	20.2	1.3	123.1
NMB (%)[b]	12.5	-17.0	16.5	-4.5

[a] T2, temperature at 2 m; RH2, relative humidity at 2 m; WS10, wind speed at 10 m; WD10, wind direction at 10 m

[b] data pairs, the number of observed and simulated data pairs; MeanObs, mean observational data; MeanSim, mean simulation results; R, correlation coefficient; MB, mean bias; RMSE, root mean square error; NMB, normalized mean bias.

Supplementary references

1. Maaß F, Elias H, Wannowius KJ. Kinetics of the oxidation of hydrogen sulfite by hydrogen peroxide in aqueous solution:: ionic strength effects and temperature dependence. *Atmospheric Environment* **33**, 4413-4419 (1999).

2. Seinfeld JH, Pandis SN. *Atmospheric chemistry and physics: from air pollution to climate change*. John Wiley & Sons (2016).

3. Cheng YF*,* et al. Reactive nitrogen chemistry in aerosol water as a source of sulfate during haze events in China. *Science Advances* **2**, e1601530 (2016).

4. Lagrange, J., Pallares, C. & Lagrange, P. Electrolyte Effects on Aqueous Atmospheric Oxidation of Sulfur-Dioxide by Ozone. *J. Geophys. Res. Atmos.* 99, 14595-14600 (1994).

5. Martin LR, Hill MW. The effect of ionic strength on the manganese catalyzed oxidation of sulfur (IV). *Atmospheric Environment* **21**, 2267-2270 (1987)

6. Martin, L. R., Hill, M. W., Tai, A. F. & Good, T. W. The Iron Catalyzed Oxidation of Sulfur(IV) in Aqueous-Solution - Differing Effects of Organics at High and Low Ph. J. *Geophys. Res. Atmos.* 96, 3085-3097 (1991).

7. Ibusuki T, Takeuchi K. Sulfur dioxide oxidation by oxygen catalyzed by mixtures of manganese (II) and iron (III) in aqueous solutions at environmental reaction conditions. *Atmospheric Environment* **21**, 1555-1560 (1987).

8. Boyce SD, Hoffmann MR. Kinetics and Mechanism of the Formation of Hydroxymethanesulfonic Acid at Low Ph. *J Phys Chem-Us* **88**, 4740-4746 (1984).
